# TPC1-Type Channels in *Physcomitrium patens*: Interaction between EF-Hands and Ca^2+^

**DOI:** 10.3390/plants11243527

**Published:** 2022-12-15

**Authors:** Franko Mérida-Quesada, Fernando Vergara-Valladares, María Eugenia Rubio-Meléndez, Naomí Hernández-Rojas, Angélica González-González, Erwan Michard, Carlos Navarro-Retamal, Ingo Dreyer

**Affiliations:** 1 Programa de Doctorado en Ciencias mención Modelado de Sistemas Químicos y Biológicos, Universidad de Talca, 2 Norte 685, Talca CL-3460000, Chile; 2 Electrical Signaling in Plants (ESP) Laboratory–Centro de Bioinformática y Simulación Molecular (CBSM), Facultad de Ingeniería, Universidad de Talca, 2 Norte 685, Talca CL-3460000, Chile; 3 Programa de Doctorado en Ciencias mención Biología Vegetal y Biotecnología, Universidad de Talca, 2 Norte 685, Talca CL-3460000, Chile; 4 Instituto de Ciencias Biológicas, Universidad de Talca, Campus Talca, Avenida Lircay, Talca CL-3460000, Chile; 5 Department of Cell Biology and Molecular Genetics, University of Maryland, College Park, MD 20742-5815, USA

**Keywords:** TPC1, vacuole, EF-domain, *Physcomitrella*, coordination of Ca^2+^

## Abstract

Two-pore channels (TPCs) are members of the superfamily of ligand-gated and voltage-sensitive ion channels in the membranes of intracellular organelles of eukaryotic cells. The evolution of ordinary plant TPC1 essentially followed a very conservative pattern, with no changes in the characteristic structural footprints of these channels, such as the cytosolic and luminal regions involved in Ca^2+^ sensing. In contrast, the genomes of mosses and liverworts encode also TPC1-like channels with larger variations at these sites (TPC1b channels). In the genome of the model plant *Physcomitrium patens* we identified nine non-redundant sequences belonging to the TPC1 channel family, two ordinary TPC1-type, and seven TPC1b-type channels. The latter show variations in critical amino acids in their EF-hands essential for Ca^2+^ sensing. To investigate the impact of these differences between TPC1 and TPC1b channels, we generated structural models of the EF-hands of PpTPC1 and PpTPC1b channels. These models were used in molecular dynamics simulations to determine the frequency with which calcium ions were present in a coordination site and also to estimate the average distance of the ions from the center of this site. Our analyses indicate that the EF-hand domains of PpTPC1b-type channels have a lower capacity to coordinate calcium ions compared with those of common TPC1-like channels.

## 1. Introduction

Two-pore channels (TPCs) are intracellular voltage- and ligand-gated cation-selective ion channels in eucaryotes. While two paralogs of this channel class are known in the human genome (TPC1s and TPC2s) [1], only TPC1-type channels exist in plants [2]. They are the molecular basis of the well-characterized non-selective slow vacuolar (SV) channels [3,4,5,6]. The prototype for studies on TPCs in plants is AtTPC1, the TPC1 from the model plant *Arabidopsis thaliana* [3,7,8,9,10].

**Figure 1:**
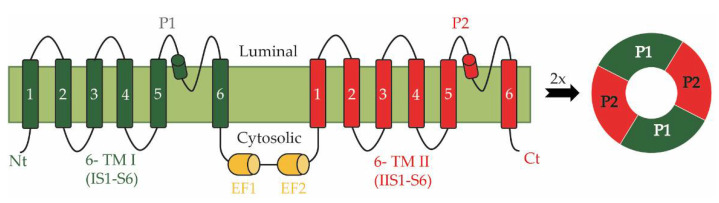
Topology of a plant Two-Pore Cation Channel 1 (TPC1). A TPC1-subunit is built of two concatenated copies of a structural 6-TM motif, each with six transmembrane domains (1–6). The cytosolic linker between them contains two EF-hands, which sense the cytosolic Ca^2+^ concentration. Two subunits assemble into a functional channel and form a central permeation pathway.

Functional TPCs are dimers in which each subunit consists of 12 transmembrane segments that partition into two non-equivalent interconnected shaker modules, each with six transmembrane regions (6-TM I and 6-TM II; Figure 1). Two subunits form a central permeation pathway [7,8] predominantly allowing in plants the passage of monovalent cations K^+^ and Na^+^ [11,12]. The cytosolic linker between 6-TM I and 6-TM II of plant TPC1s usually contains two Ca^2+^-binding EF-hands that are involved in the regulation of channel activity by cytosolic Ca^2+^ [13,14,15,16]. In addition to the EF-hands, which sense [Ca^2+^]_cyt_, the activity of TPC1s (SV channels) is also regulated by vacuolar Ca^2+^, involving residues at the luminal side of the channel [17,18,19,20,21,22,23].

The EF-hand domain comprises the EF1 and EF2-hands, each consisting of two alpha helices (E and F) and a loop. The residues that form the calcium-binding site are mainly located in the loop and are usually negatively charged or hydrophobic. The interactions that stabilize calcium binding in EF-hands are hydrogen bonds between the cation (Ca^2+^) and negatively charged residues or carboxyl groups of the calcium-oriented backbone [24]. In the case of AtTPC1, calcium binding in the EF1-hand has been described to play mainly a structural role, while the EF2-hand modulates channel activation by triggering a conformational change that opens the passage of ions through the pore [8,13].

Interestingly, during the evolution of land plants, the characteristic structural features of the cytosolic and luminal regions involved in Ca^2+^ perception of plant TPC1s have been preserved without significant changes and are found in TPC1s from charophytes to angiosperms [17]. In mosses and liverworts, however, there is another clade of the TPC1-type (TPC1b) [17,25], which has been lost in vascular plants and hornworts. Channels of the TPC1b-type seem to almost completely lack the typical EF-hand domain architecture, at least if automatic domain predictions are considered (Table S1). Although these channels still share the general structural architecture of TPC1, the amino acids of the Ca^2+^ binding site in the EF-hands have a higher proportion of positively charged residues. This suggests that calcium binding in the EF-hands of these TPC1b-type channels has a reduced number of interactions, which may cause a weakening of the binding sites. These observations raise the question of whether these EF-hand-like structures can still coordinate Ca^2+^ ions.

To address the issue of Ca^2+^-binding in EF-hand-like structures of TPC1b-type channels, we identified and analyzed the sequences of TPC1-like channels from the model-moss *Physcomitrium patens* (synonym: *Physcomitrella patens*; *P. patens*), and generated molecular homology models of their putative EF-hands. These were then used to investigate the interaction with Ca^2+^ ions in molecular dynamics simulations.

## 2. Results

### 2.1. Nine TPC1-Like Channels in P. patens

**Figure 2:**
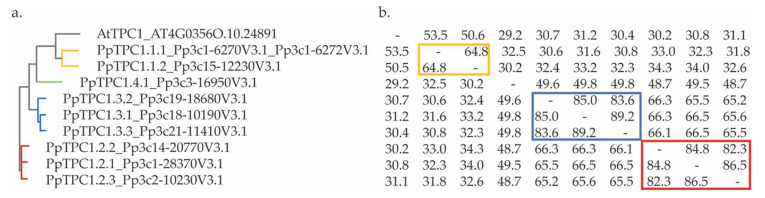
Ancestry and homology analysis of TPC1-like sequences from *P. patens*. (**a**). Cladogram of the TPC1 sequences in *A. thaliana* and *P. patens*; (**b**) Percent identity matrix corresponding to the alignment of the TPC1 channel sequences of *A. thaliana and P. patens*. The colors indicate cluster 1 (yellow), cluster 2 (red), cluster 3 (blue), and cluster 4 (green).

To identify TPC1-like channels in *P. patens* we screened the proteome of this moss in a BLAST search with the sequence of AtTPC1 as query using the Phytozome database (https://phytozome-next.jgi.doe.gov/; accessed on 30 August 2021). The initial 46 hits were manually checked for redundancies, which finally resulted in nine different sequences for TPC1-like proteins that were presumably full-length. Next, an alignment of these sequences with the AtTPC1 sequence was performed followed by clustering to identify the relationships between them. Analysis of the cladogram and identity matrix revealed that the sequences could be assigned to four main clusters. The first cluster consisted of two sequences (PpTPC1.1.1_Pp3c1-6270V3.1, which coded for an identical protein as Pp3c1-6272V3.1, and PpTPC1.1.2_Pp3c15-12230V3.1), which shared 65% identity and clustered with AtTPC1. The second cluster comprised three sequences (PpTPC1.2.1_Pp3c1-28370V3.1, PpTPC1.2.2_Pp3c14-20770V3.1, and PpTPC1.2.3_Pp3c2-10230V3.1), which shared 82–86% identity. The third cluster was also formed by three sequences (PpTPC1.3.1_Pp3c18-10190V3.1, PpTPC1.3.2_Pp3c19-18680V3.1, and PpTPC1.3.3_Pp3c21-11410V3.1), with the percentages of identity ranging from 83–89%. Finally, in the fourth cluster, only one sequence was observed (PpTPC1.4.1_Pp3c3-16950V3.1), which had a low percentage of identity (<50%) with the other sequences (Figure 2).

### 2.2. Analysis of the Identity and Similarity of the EF-Hands of AtTPC1 and PpTPC1s

**Figure 3:**
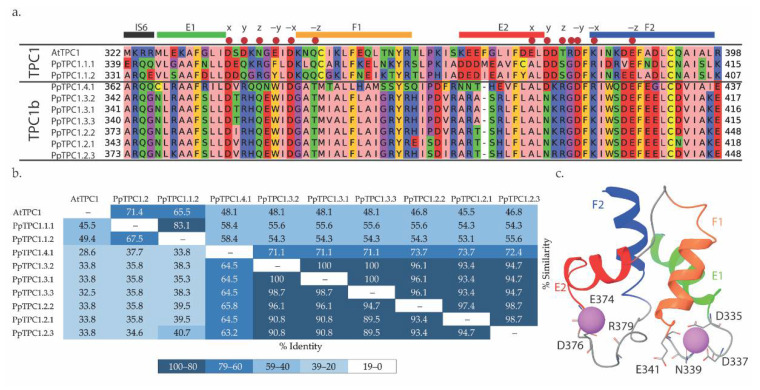
EF-hand sequences of TPC1-type channels from *A. thaliana* and *P. patens*. (**a**) Sequence alignment, separating between TPC1 and TPC1b. The TPC1 group consisted of AtTPC1 and the PpTPC1-sequences from cluster 1. The TPC1b group consisted of the PpTPC1-sequences of clusters 2–4. For each sequence, the residue numbers of the beginning and end of the EF-hand domains are shown. Zappo staining was used to distinguish the characteristics of the residues: negatively charged (red), positively charged (blue), polar (green), aromatic (mustard), hydrophobic (pink), cysteine (yellow), and proline and glycine (purple). Colored bars on top of the alignment indicate the alpha-helix structure of the EF-hands of AtTPC1. Red dots indicate residues reported for calcium-binding in the AtTPC1 EF-hands. (**b**) Identity/similarity matrix of the EF-hand sequences of TPC1 channels from *A. thaliana* and *P. patens*. Numbers above the diagonal represent the percentage of similarity and those below the diagonal represent the percentage of identity. (**c**) Fragment of the AtTPC1 channel (PDBID:6E1N) corresponding to the EF-hands. The alpha helices E and F are color-coded as on the top of panel (**a**). Residues reported for Ca^2+^ binding that form hydrogen bonds in this crystal are highlighted.

We then identified the putative EF-hand regions in the alignment of the *P. patens* TPC1 sequences (Figure S1). The EF-hand sections were extracted, and a new alignment was made to check which regions were most similar to AtTPC1 and determine the level of conservation in this domain (Figure 3). Based on the pairwise identity values, the EF-hand sequences could be split into two groups: the first with EF-hands of AtTPC1, PpTPC1.1.1, and PpTPC1.1.2, and the second with those of the other *P. patens* channels. The within-group sequences had identities of >45%, whereas between-groups, the identity of the EF-hand sequences was <45% (Figure 3b). The TPC1s of the second group have been previously identified to belong to a moss/liverwort-specific sub-clade (TPC1b) [17].

To further analyze the relationships between the identified EF-hand domains and other proteins of this huge protein family [26], we first made Blast searches (https://blast.ncbi.nlm.nih.gov/Blast.cgi; accessed on 1 December 2022) with the isolated EF-hands of the TPC1b-type channels. A run against all non-redundant protein sequences in the NCBI database revealed hits only for other mosses (*Ceratodon*, *Sphagnum*) and liverworts (*Marchantia*), indicating further the correlation of these specific structures with the Setaphyte branch in the plant kingdom [17]. In contrast, when the isolated EF-hands of the ordinary TPC1-type channels PpTPC1.1.1 and PpTPC1.1.2 were used as queries, the Blast searches identified TPC1-like channels from bryophytes to angiosperms. When we compared the tandem EF-hand domains of TPC1-like channels from streptophyte algae, hornworts, liverworts, mosses, ferns, and angiosperms, we obtained the same clustering into TPC1/TPC1b-clades as was observed for the full-length TPC1-like channel proteins (Table S1, Figure S2). Next, we isolated 484 EF-hand domains from proteins of *A. thaliana* [27] (Table S2) and clustered them with the 18 EF-hand domains from the *P. patens* TPC1-like channels and the two EF-hand domains from AtTPC1 (Figure S3). All TPC1-originating EF-hand domains clustered together, regardless of whether they came from ordinary TPC1 or TPC1b-like channels. Taken together, these analyses suggested that the EF-hands in TPC1/TPC1b-like channels had a common origin, despite their differences.

Based on the domains of the EF-hands reported for AtTPC1 (PDBID:6E1N) [28], the essential amino acids involved in the active sites of Ca^2+^ binding could be identified (Figure 3c; Table S3). Comparison of ATPC1 with the PpTPC1-sequences revealed relevant changes in the active sites of the EF-hands of TPC1b-type channels, especially in the EF2-hand (Figure 3a). There, the TPC1b-type channels had fewer negative residues and more positive residues. Thus, it was tempting to speculate that the change in the chemical environment of the active ion coordination sites may have affected Ca^2+^ interaction in the EF2-hands of these channels.

Next, we investigated in further detail the structural aspects of the Ca^2+^-binding sites in the EF-hands. To keep this approach feasible, we selected four of the 10 TPC1-like channels. Besides AtTPC1, we chose PpTPC1.1.1 to represent the common TPC1s, and PpTPC1.2.1 and PpTPC1.4.1 to represent the TPC1b-clade. The high identity (>89%) of the EF-hands in the TPC1-like channels of clusters 2 and 3 emboldened us to select only one representative of these six sequences. After this selection, we generated models of the EF-hands for AtTPC1, PpTPC1.1.1, PpTPC1.2.1, and PpTPC1.4.1.

### 2.3. Electrostatic Surface of EF-Hands of TPC1- and TPC1b-Type Channels

**Figure 4:**
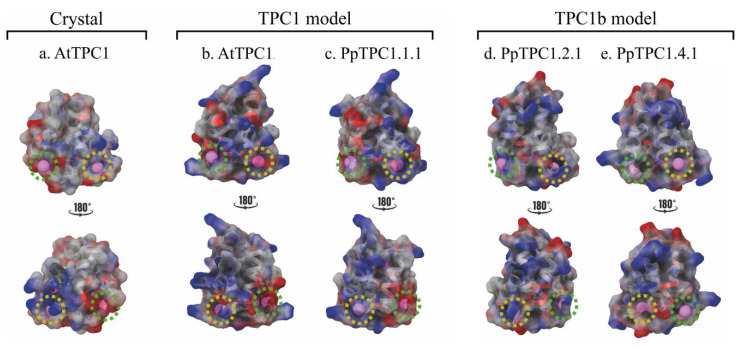
Electrostatic surface of the EF-hands. The electrostatic surface images (**a**) of the AtTPC1 crystal and the models for (**b**) AtTPC1, (**c**) PpTPC1.1.1, (**d**) PpTPC1.2.1, and (**e**) PpTPC1.4.1 are shown in front (**upper** panel) and dorsal view (**lower** panel). The red regions refer to areas with negatively charged residues; the blue regions refer to areas with positively charged residues; the grey regions refer to hydrophobic areas. The green and yellow dotted circles refer to the Ca^2+^ binding sites of the EF1-hand and EF2-hand, respectively.

At first, we displayed the electrostatic surface of the created models (Figure 4, front and back). For comparison, we added the fragment of the EF-hands of the crystal of AtTPC1 (PDBID:6E1N). Like in the AtTPC1 crystal structure, the Ca^2+^ binding sites in the models of the EF-hands of AtTPC1 and PpTPC1.1.1 were also electronegative (Figure 4a–c). This result correlated well with a higher presence of negative amino acids in the chemical environment of the active sites of the EF-hands (Figure 3a), which is favorable for the coordination of calcium ions in this region. In contrast, the homologous sites in the EF-hands of TPC1b-type channels (PpTPC1.2.1 and PpTPC1.4.1) showed a lower intensity of the red color (Figure 4d,e). This finding correlated with a higher proportion of positively charged and hydrophobic residues in the putative Ca^2+^-binding sites (Figure 3a), which might be unfavorable for the coordination of calcium ions.

### 2.4. Analysis of Molecular Dynamics

**Figure 5:**
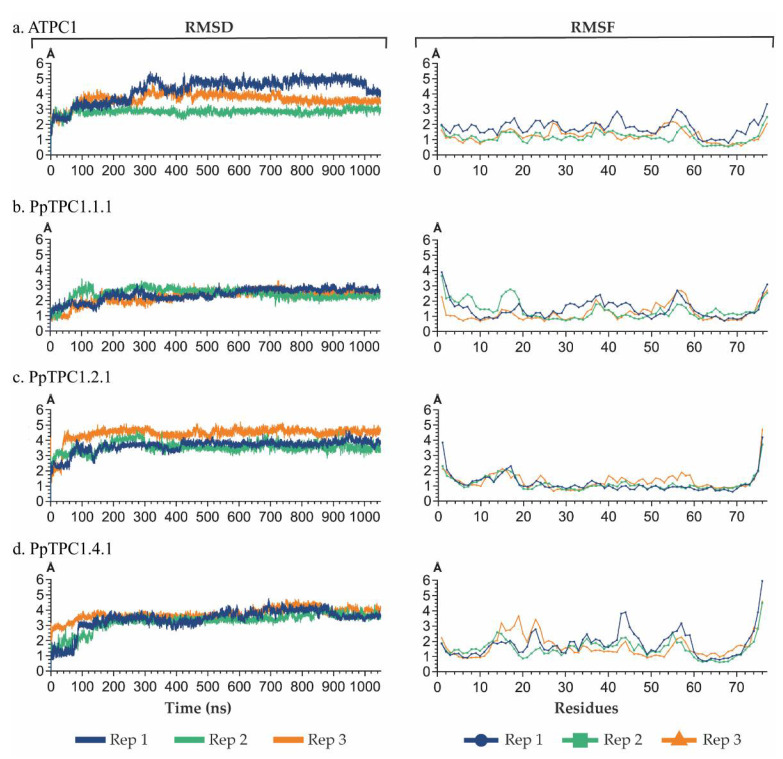
Analysis of the root-mean-square deviation (RMSD) and root-mean-square fluctuation (RMSF) of the MD simulations of the EF-hand domains from (**a**) AtTPC1, (**b**) PpTPC1.1.1, (**c**) TPC1.2.1, and (**d**) TPC1.4.1. In both RMSD and RMSF, the blue, green, and orange curves represented the three replicates of the molecular dynamics simulations performed for each model, with a duration of 1050 ns for each. The RMSD and RMSF analyses were performed using the first molecular dynamics frame as a reference.

The models were the starting points for molecular dynamics simulations. For each model, a time interval of 1050 ns was simulated in three replicas. After 50 ns of relaxation, 1000 ns of molecular dynamics were run to follow the interaction between the Ca^2+^ ions and the EF-hands. Root-mean-square deviation (RMSD) showed that the last 500 ns of each dynamic showed greater stability, with no fluctuations greater than 1 Å in the RMSD plot (Figure 5). On the other hand, root-mean-square fluctuation (RMSF) indicated that, generally, the highest values in the replicas were found between residues 14 and 22, 37 and 42, and 55 and 63, corresponding to the loops of the EF-hands, which had greater flexibility than the alpha helices (Figure 5). In addition, it was observed that the RMSF value increased in the residues at the ends, where part of the secondary structure was also lost.

**Figure 6:**
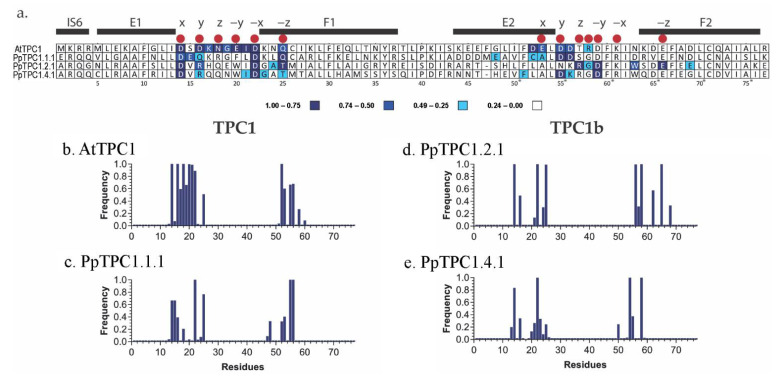
Relative frequencies of the interaction of the residues with Ca^2+^. Calculating the interaction frequencies with Ca^2+^ in molecular dynamics simulations for each model was performed with a cutoff of 5 Å to calcium. The frequencies were estimated from a total of 1500 ns of molecular dynamics, corresponding to the last 500 ns, with three replicates per model. (**a**) Alignment of amino acid sequences per model; the blue color shows the frequency of interaction of each residue with the Ca^2+^ ion within at a distance of up to 5 Å. The higher the frequency, the higher the probability of being part of the Ca^2+^-binding site. Alpha helices are represented by black bars, and residues reported as part of the calcium-binding site in the AtTPC1 crystal structure are represented by red dots. The numbering corresponds to the consecutive numbering used in the models. Please note the gap at position 47 of the alignment of PpTPC1.2.1 and PpTPC1.4.1. To cope with this gap, for values ≥ 47, one position needs to be subtracted for these two channels (indicated by an asterisk *). (**b**–**e**) Relative interaction frequency of the residues with Ca^2+^: (**b**) AtTPC1 and (**c**) PpTPC1.1.1 correspond to the EF-hand models of the TPC1 type channels, and (**d**) PpTPC1.2.1 and (**e**) PpTPC1.4.1 correspond to the TPC1b-type channels.

The relative stability of the EF-hand models allowed the interaction of each residue with the initially positioned Ca^2+^ ion over time to be determined. Frames were taken every 0.1 ns, and the minimum distance between each hydrogen-free residue and calcium was considered. Residues found within a distance of 5 Å to the calcium were considered more likely to form hydrogen bonds with Ca^2+^. In addition, in the environment where calcium binds, the distance distribution for the residues was recorded by evaluating the distance between the center of mass and the nearest calcium. The reason for choosing the distance between the centers of mass of the residues was to better represent the position of the entire residue with respect to the calcium in the molecular dynamics simulations. On the other hand, a search for interactions at distances less than 5 Å between residues and calcium would have corresponded to the assumption that it was not the entire residue but only those atoms that were closest to the calcium ion that would participate in the interaction. The distance of 5 Å was chosen because, in that vicinity, the Ca^2+^ ion could establish hydrogen bonds with the side chains of charged or polar residues or with the carboxyl groups in the backbone (Figure 6).

**Figure 7:**
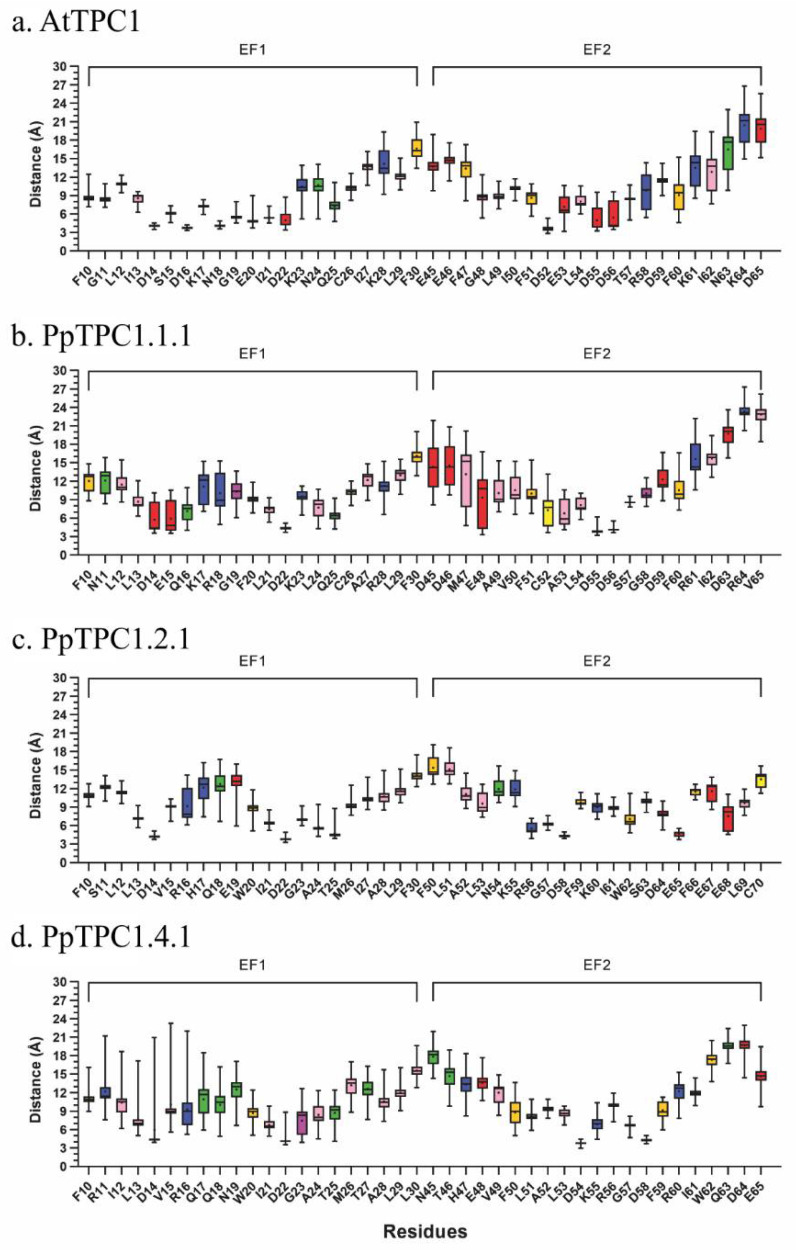
Distribution of distances between each residue and Ca^2+^. Boxplots show the distances between calcium and the center of mass of the residues in the vicinity of the EF-hand binding sites from (**a**) AtTPC1, (**b**) PpTPC1.1.1, (**c**) PpTPC1.2.1, and (**d**) PpTPC1.4.1. Zappo staining was used to distinguish the characteristics of the residues: red: negatively charged, blue: positively charged, green: polar, ochre: aromatic, pink: hydrophobic, yellow: cysteine, and magenta: proline and glycine. For both the EF1-hand and the EF2-hand, the box amplitude indicated the dispersion of the distances, where the greater dispersion indicated a more significant absence of Ca^2+^ binding and vice versa.

The simulations with the AtTPC1 model confirmed six residues (D14, D16, N18, E20, D22, and Q25) in the EF1-hand, which were reported in the crystal structure to be part of the Ca^2+^-binding site. The results for the EF2-hand, however, were different. Here only two more frequently approached residues coincided with the proposed Ca^2+^-binding site. Instead, a pattern of the residues D52, E53, D55, and D56 was most frequently visited in this EF-hand (Figure 6a,b). We also calculated the average distance between the Ca^2+^ ion and the residues (Figure 7). These values were generally larger for the EF2-hand than for the EF1-hand. Average distances of more than 9 Å did not allow the state of coordination of calcium with these residues to be reached during the molecular dynamics simulations (Figure 7a).

In contrast to AtTPC1, the model of the EF-hands of PpTPC1.1.1 showed an overall lower number of interactions with calcium. Nevertheless, some exposed residues could also be pinpointed here. These were, in particular, D22, Q25, D14, and E15 in the EF1-hand, but only D55 and D56 in the EF2-hand (Figure 6c). Nevertheless, both residues established hydrogen bonds with a distance to the Ca^2+^ ion of less than 5 Å throughout the trajectory (Figure 7b).

In the model of the EF-hands of the TPC1b-type channel PpTPC1.2.1, we identified more frequent Ca^2+^ interactions with residues D14, D22, and T25 in the EF1-hand, and R56, D58, and E65 in the EF2-hand (Figure 6d). The average distance between these residues and Ca^2+^ was low enough that hydrogen bonds could be formed (Figure 7c). It is noteworthy that these residues are highly conserved in *P. patens* TPC1b-type channels.

Despite this conservation, however, we found only very few interactions with Ca^2+^ in the model of the PpTPC1.4.1 EF-hands. In the EF1-hand, there were residues D14 and D22 as in PpTPC1.2.1, while in the EF2-hand, there were residues D54 and D58 (Figure 6e).

## 3. Discussion

Plant two-pore cation channels 1 (TPC1) are regulated by cytosolic Ca^2+^ employing structures called EF-hands that are located in the linker between the two concatenated shaker-like transmembrane domains 6-TM I and 6-TM II. Mutagenesis experiments on AtTPC1 from *Arabidopsis thaliana* have identified amino acids that are crucial for EF-hand function [8,13]. The physiological significance of these residues is that they modulate channel opening due to a conformational change that occurs in the respective EF-hand when a calcium ion is coordinated [29,30,31,32,33,34,35]. Sequence analyses have indicated that the EF-hands of some TPC1s from *P. patens*, other mosses, and the liverwort *Marchantia polymorpha* differ from those of AtTPC1 (Figure S2 and Figure 3a) [17], causing a different chemical environment of the active sites (Table S3) [7,8,36,37,38]. Therefore, in this study, we modeled the EF-hands of TPC1-type channels from *P. patens* and analyzed the electrostatic surfaces in the obtained models. We observed, in particular, differences between the systems for AtTPC1 and PpTPC1b-type channels. The lower electronegativity in the active sites of PpTPC1.2.1 and PpTPC1.4.1 (TPC1b-type) goes hand in hand with the predominance of positively charged amino acids in these sites. In turn, the presence of positive residues did not prevent the formation of a Ca^2+^-binding pocket by negatively charged, polar residues and presumably the participation of carboxyls. Indeed, the positively charged side chains showed an orientation toward the outside and did not interfere significantly with the Ca^2+^ binding. This feature allowed Ca^2+^ to bind to the EF-hand models of both TPC1 and TPC1b and these interactions were preserved throughout the molecular dynamics simulations. Although this configuration of the EF-hand binding site in TPC1b was favorable, they generally exerted a lower Ca^2+^ ion coordination capacity in this region when compared with the EF-hands of AtTPC1.

Residue D14 (D335 in AtTPC1) was associated with a structural role in Ca^2+^ binding in the EF1-hand of AtTPC1. Together with D22, they were the only negatively charged residues conserved in the EF1-hands of PpTPC1- and PpTPC1b-type channels (Figure 3a), giving them greater importance for calcium coordination. Additionally, at position 25 of the EF-hand sequence alignment, a polar residue involved in calcium binding was conserved, this being Q25 for the TPC1-type EF-hand models and T25 for the TPC1b-type EF-hand models (Figure 3a). In the molecular dynamics simulations, we observed stable interactions of these residues with Ca^2+^ ions, corresponding to the formation of hydrogen bonds (Figure 6a). Although the EF-hands of the TPC1b-type channels had a higher proportion of negatively charged residues than those of TPC1-type channels, this did not disturb the stable calcium binding to the polar residue throughout the molecular dynamics simulations (Figure 6a,d,e). Interestingly, positively charged residues close to those that establish stable interactions with Ca^2+^ showed an average distance greater than 9 Å from the ion (Figure 7). This finding further supported the hypothesis that the side chains of these residues were oriented outward from the binding site, favoring a more electronegative environment.

The molecular dynamics simulations of the AtTPC1 model indicated that most of the residues that evidenced hydrogen bonds in the crystal (PDBID:6E1N) conserved these interactions throughout the dynamics, demonstrating a greater amount of residues involved in calcium binding in the EF1-hand (Figure 6a,b). The confirmation of the bonds identified in the crystal was evidence of their stability and strongly supported the effectiveness of the methods employed in our study for explaining the dynamic features of the calcium-binding sites.

Using alanine mutagenesis, Guo et al. [8] reported the importance of residue D55 (D376 in AtTPC1) for AtTPC1 channel activity. In this study, 86% of the PpTPC1b-type channels, represented by PpTPC1.2.1 (Figure 3a), showed a polar asparagine (N) instead of a negatively charged aspartic acid (D) at this position. As a consequence, no interactions between this residue (N54) and calcium were found in PpTPC1.2.1 (Figure 7c). In contrast, PpTPC1.4.1 did have the D55 residue and showed EF2-hand interactions more similar to those observed with AtTCP1 than those of PpTPC1.2.1 (Figure 6).

Rather robust calcium hydrogen bonds in the EF2-hand of PpTPC1.2.1 were observed for the two residues D58 and E65 that were conserved in the other three investigated channels (Figure 6a,d). Nevertheless, in the other three models, these residues did not play a role in Ca^2+^ coordination. Instead, interactions were established with residues close to the initial position in the EF2-hand where the Ca^2+^ ion was located. In summary, more residues interacting with Ca^2+^ were observed in AtTPC1 than in PpTPC1.2.1, suggesting that PpTPC1b-type channels could coordinate calcium in both EF-hands but to a lesser extent than observed in AtTPC1.

From an evolutionary point of view, the TPC1s from *P. patens* are more ancient than AtTPC1 (Figure S2a). In direct comparison, Ca^2+^ binding to the EF-hands of AtTPC1 can be considered already optimized. TPC1s from mosses and liverworts could therefore be seen as an experimental laboratory of evolution, where different mechanisms are still being tried out. This would at least explain the high number of such channel-encoding genes in the genomes and the collapse of this diversity during further plant evolution. Future studies need to uncover the physiological role of the many TPC1s in mosses. Could it be that they perform functions that TPC1s in vascular plants do not normally assume?

In Arabidopsis, AtTPC1 forms the slow vacuolar (SV) channel [3]. Nevertheless, SV channels have also been characterized in non-vascular mosses and liverworts [39,40,41,42]. *Marchantia polymorpha* is a representative of liverworts, which are regarded as pioneer plants that colonized the land around 500 million years ago. The genome of *M. polymorpha* encodes for three TPC1-like channels, one of the ordinary type and two of the TPC1b-type [17,25]. Interestingly, SV channel activity was abolished in the loss-of-function mutant of the ordinary TPC1-type, while single and double mutants in the TPC1b-types exhibited SV channel activity similar to the wild type. This observation further fuels the hypothesis that TPC1b-type channels may be different from SV channels.

## 4. Materials and Methods

### 4.1. TPC1 Sequences of Physcomitrium patens

The sequences of TPC1-like channels in the *P. patens* proteome were retrieved from the Phytozome database (http://www.phytozome.net/; accessed on 30 August 2021) using the *Arabidopsis thaliana* TPC1 sequence as a template. This process resulted in about 46 similar sequences; this set was then screened for duplicate and incomplete sequences using the Clustal Omega multiple sequence alignment tool (https://www.ebi.ac.uk/; accessed on 30 August 2021). After eliminating redundant sequences and sequences with insertions, deletions, or putative splicing variants, nine sequences remained. These were compared with the AtTPC1 sequence for similarities, again using the Clustal Omega multiple sequence alignment tool (https://www.ebi.ac.uk/; accessed on 20 September 2021) resulting in four clusters.

### 4.2. EF-Hand Domain Identification and Clustering

We identified all domains present in the proteins of interest with Prosite local or using the InterPro Web-Server (https://www.ebi.ac.uk/interpro/; accessed in the period from May 2022 to December 2022) and selected all sequences with EF-hands hits of a *p*-value ≤ 1 × 10^−5^ (*p* ≤ 0.00001). For several *P. patens* TPC1b-type channels, the automatic prediction of EF-hand domains failed. In these cases, they were extracted from the sequence alignment with AtTPC1. For phylogenetic analyses shown in Figure S2, we used the paired EF domains, while for cluster analyses shown in Figure S3, we used all separated domains because not all proteins had paired EF domains. Sequence alignment was performed with Clustal Omega. Mega software v11.1 (https://www.megasoftware.net/; accessed several times in November/December 2022) was then used to perform phylogenetic analysis using the maximum parsimony statistical method, with a boostrap phylogeny test with 1000 replications.

### 4.3. Model Building

The sequences of the EF-hands from AtTPC1 (AT4G03560.1:322-398) and those from PpTPC1.1.1 (Pp3c1-6270v3.1:339-415), PpTPC1.2.1 (Pp3c1-28370v3.1:343-418), and PpTPC1.4.1 (Pp3c3-16950v3.1:362-437) were used. Sequences from PpTPC1-cluster 3 were not considered as they exhibited 97% similarity to the sequence of the PpTPC1.2.1 EF-hands. The crystal of AtTPC1 (PDBID:6E1N) was selected as a template for its resolution and retaining Ca^2+^ cations in its EF-hands. The ColabFold tool [43] was used to create the models, and five models were generated for each of the four sequences, from which the best one was selected using the pLDDT score according to Mariani et al. [44] (Figure S4). Finally, two Ca^2+^ ions were included on each model, at the same positions where they were localized in the AtTPC1 crystal.

### 4.4. System Preparation

Systems were prepared with the Maestro-Schrödinger suite (Schrodinger, LLC, New York, NY, USA, 2020). For each model, a 55 Å-sided orthorhombic box was generated. The systems were solvated using the SPC water model [45]. To mimic biological conditions, 150 mM of KCl was added, excluding ion and salt placement within 5 Å of the protein. The OPLS3e force field was used.

### 4.5. Molecular Simulation

For the MD simulations, the Desmond module (Schrodinger, LLC, New York, NY, USA, 2020) was used. At first, Desmond default relaxation protocol of the system was performed to relax the system. Later, for equilibration purposes, 50 ns of MD simulations were performed applying a 50 kcal × mol^−1^ × Å^−2^ constraint on the alpha carbon atoms of the residues at each end of the models to keep the stecheometry of the EF-hands in the absence of the rest of the protein. Likewise, a 50 kcal × mol^−1^ × Å^−2^ constraint was applied to the Ca^2+^ cations, in order to allow the EF-hands to accommodate each Ca^2+^ ion. For the production run, 1000 ns of molecular dynamics were performed, only retaining the constraint on the alpha carbons of the residues at each end of the models. For all simulations, the temperature and pressure were kept constant at 300 K and 1.01325 bar respectively with semi-isotropic conditions and an NPT assembly. Finally, all these calculations were performed in triplicate for each system.

### 4.6. Trajectory Analysis

VMD software [46] was used for molecular dynamics trajectory analysis. RMSD and RMSF calculations considered the entire trajectory of each system (1050 ns). The last 500 ns of the trajectory was considered with a cutoff of 5 Å to count the frequency of Ca^2+^ interactions per residue in the EF-hands loop.

## Data Availability

All data are included in the manuscript. Additional information is available upon request from the corresponding authors.

## Supplementary Materials

The following supporting information can be downloaded at: https://www.mdpi.com/article/10.3390/plants11243527/s1, Figure S1: Alignment of the full-length sequences of the TPC1s from *A. thaliana* and *P. patens*; Figure S2: Phylogenetic analysis of tandem EF-domains from TPC1-like channels; Figure S3: Comparison of EF-hand domains found in proteins from *A. thaliana* and in TPC1-like channels from *P. patens*; Figure S4: Residual confidence plot of the EF-hand models; Table S1: Automatic identification of EF-hand domains in plant TPC1-like channels from angiosperms, ferns, mosses, liverworts, hornworts, and streptophyte algae; Table S2: EF-hand domains in proteins from *A. thaliana*, and in TPC1s from *P. patens*; Table S3: EF-hand residues forming hydrogen bonds with calcium found in reported crystals of AtTPC1.
