# Supplementary material for: TPC1-Type Channels in Physcomitrium patens: Interaction between EF-Hands and Ca2+
Source: Plants (Basel). 2022 Dec 15;11(24):3527. doi: 10.3390/plants11243527 (PMC9783492; doi:10.3390/plants11243527)
Supplement: Supplementary file 1 [file plants-11-03527-s001.zip › Merida_et_al_Supplementary_Material.pdf]

plants

Supplementary Material

TPC1-type channels in *Physcomitrium patens*: interaction between EF-hands and Ca^{2+}

Mérida-Quesada F., Vergara-Valladares F., Rubio-Meléndez M.E., Hernández-Rojas N., González-González A., Michard E., Navarro-Retamal C., Dreyer I.

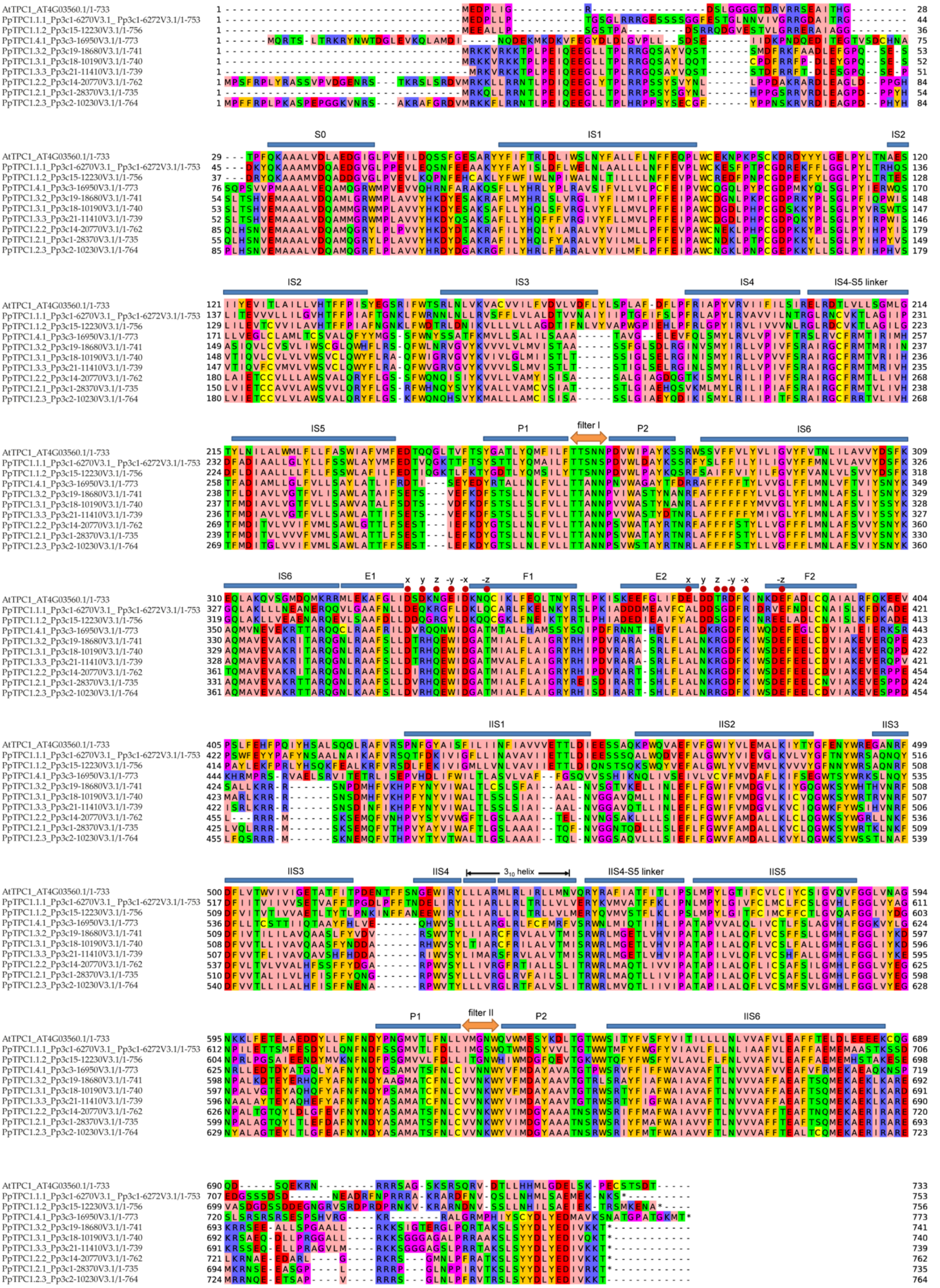

Figure S1. Alignment of the full-length sequences of the TPC1s from *A. thaliana* and *P. patens*. Alpha helices are represented by blue bars, and the residues reported as part of the calcium-binding site in the AtTPC1 crystal structure are represented by red dots. The Zappo staining was used to distinguish the characteristics of de residues.

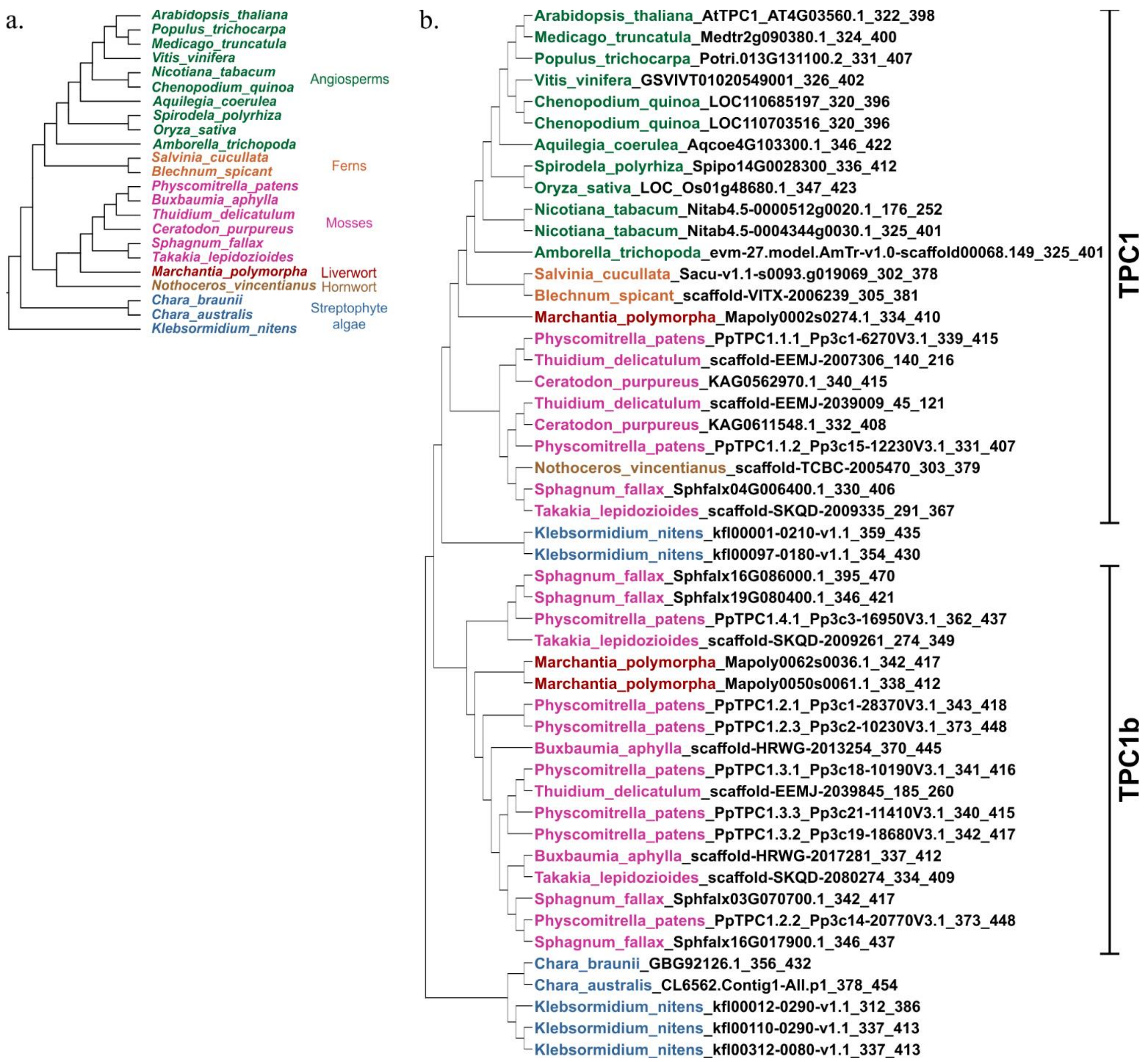

Figure S2. Phylogenetic analysis of tandem EF-domains from TPC1-like channels. (**a**) Species tree of plant species. Species affiliation to the plant family is color coded. (**b**) Phylogenetic tree of tandem EF-domains from TPC1-like channels. The separate group, found only in mosses and liverworts, gathers the tandem EF-hand-like domains of TPC1b-type channels.

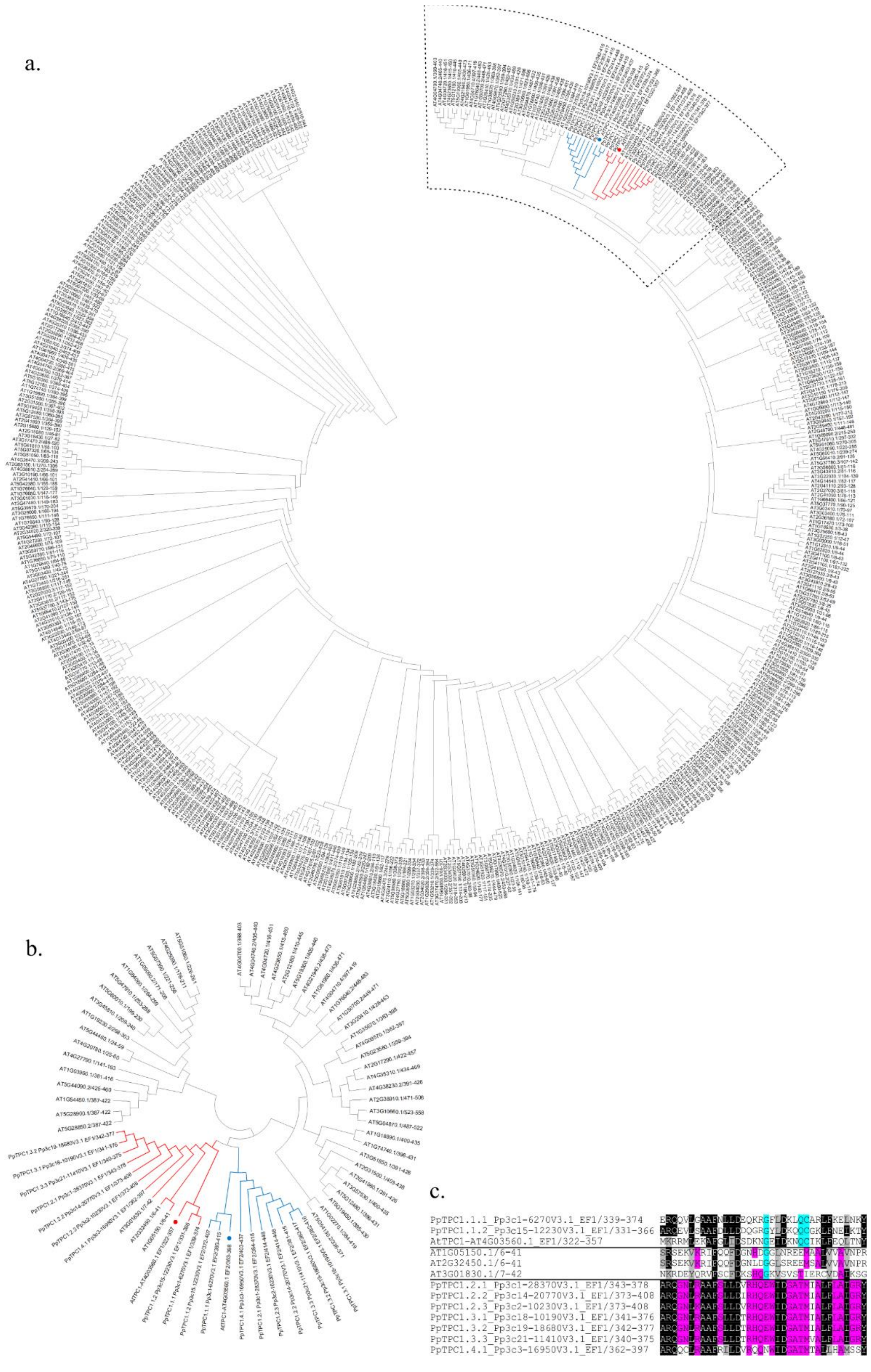

Figure S3. Comparison of EF-hand domains found in proteins from *A. thaliana* and in TPC1-like channels from *P. patens*. (**a**) Cladogram of 486 EF-hand domains found in proteins from *A. thaliana* and 18 found in TPC1-like channels from *P. patens*. A high-resolution version of this figure is provided as separate file. (**b**) Close-up view of the region marked in (a). (**c**) Sequence alignment of the sequences marked in red in (a) and (b). Sites specific for TPC1b-type channels are highlighted in magenta, sites specific for ordinary TPC1-type channels are highlighted in cyan.

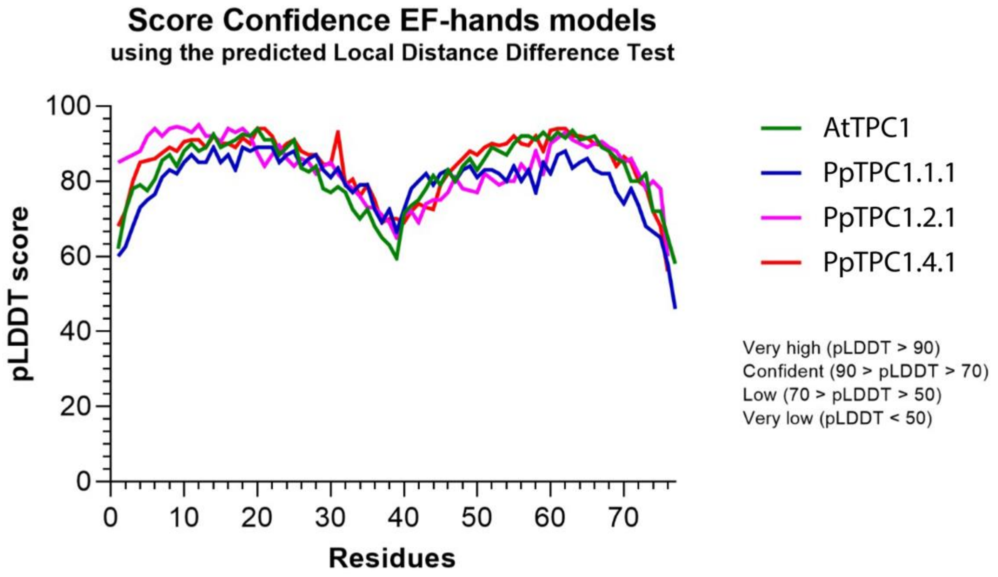

Figure S4. **Residual confidence plot of the EF-hand models.** Using Colabfold to generate the models, a score associated with the quality of the model is also obtained using a per-residue confidence metric called the predicted local distance difference test (pLDDT) on a scale of 0 to 100. In this sense, our backbone orientation and side chains valuation are considered with a pLDDT >70%.

Table S1: Automatic identification of EF-hand domains in plant TPC1-like channels from angiosperms, ferns, mosses, liverworts, hornworts and streptophyte algae. TPC1-like protein sequences were screened for functional domains using the InterPro Web-Server (**https://www.ebi.ac.uk/interpro/**; accessed the last time on 12 December 2022). The identified IDs (cd00051, IPR002048, IPR011992, IPR018247, PF13499, PS00018, PS50222, SM00054, SSF47473) indicate that TPC1-like channels have EF-hand_2 motifs.

	**Species/Gene name/Accession number**	**EF-hand domains identified with https://www.ebi.ac.uk/interpro/**	**TPC1 / TPC1b**
Angiosperms	*Arabidopsis_thaliana*_AtTPC1_AT4G03560.1	IPR002048; cd00051; PS50222; SM00054; IPR011992; SSF47473	TPC1
	*Populus_trichocarpa*_Potri.013G131100.2	IPR002048; SM00054; PS50222; cd00051; IPR011992; SSF47473	TPC1
	*Medicago_truncatula*_Medtr2g090380.1	IPR002048; PS50222; IPR011992; SSF47473	TPC1
	*Vitis_vinifera*_GSVIVT01020549001	IPR002048; PS50222; IPR011992; SSF47473	TPC1
	*Nicotiana_tabacum*_Nitab4.5-0000512g0020.1	IPR002048; cd00051; SM00054; PS50222; IPR011992; SSF47473	TPC1
	*Nicotiana_tabacum*_Nitab4.5-0004344g0030.1	IPR002048; cd00051; PS50222; SM00054; IPR011992; SSF47473	TPC1
	*Chenopodium_quinoa*_LOC110685197	IPR002048; PS50222; IPR011992; SSF47473	TPC1
	*Chenopodium_quinoa*_LOC110703516	IPR002048; PS50222; IPR011992; SSF47473	TPC1
	*Aquilegia_coerulea*_Aqcoe4G103300.1	IPR011992; SSF47473	TPC1
	*Spirodela_polyrhiza*_Spipo14G0028300	IPR002048; PS50222; cd00051; IPR011992; SSF47473	TPC1
	*Oryza_sativa*_LOC_Os01g48680.1	IPR002048; SM00054; PS50222; IPR011992; SSF47473	TPC1
	*Amborella_trichopoda*_evm-27.model.AmTr-v1.0-scaffold00068.149	IPR011992; SSF47473	TPC1
Ferns	*Salvinia_cucullata*_Sacu-v1.1-s0093.g019069	IPR002048; PS50222; IPR011992; SSF47473	TPC1
	*Blechnum_spicant*_scaffold-VITX-2006239	IPR002048; PS50222; IPR011992; SSF47473	TPC1
Mo	*Physcomitrium_patens*_PpTPC1.1.1_Pp3c1-6270V3.1	IPR011992; SSF47473	TPC1

	*Physcomitrium_patens*_PpTPC1.1.2_Pp3c15-12230V3.1	IPR002048; PS50222; IPR011992; SSF47473	TPC1
	*Physcomitrium_patens*_PpTPC1.2.1_Pp3c1-28370V3.1	No EF domain identified	TPC1b
	*Physcomitrium_patens*_PpTPC1.2.2_Pp3c14-20770V3.1	No EF domain identified	TPC1b
	*Physcomitrium_patens*_PpTPC1.2.3_Pp3c2-10230V3.1	No EF domain identified	TPC1b
	*Physcomitrium_patens*_PpTPC1.3.1_Pp3c18-10190V3.1	No EF domain identified	TPC1b
	*Physcomitrium_patens*_PpTPC1.3.2_Pp3c19-18680V3.1	No EF domain identified	TPC1b
	*Physcomitrium_patens*_PpTPC1.3.3_Pp3c21-11410V3.1	No EF domain identified	TPC1b
	*Physcomitrium_patens*_PpTPC1.4.1_Pp3c3-16950V3.1	No EF domain identified	TPC1b
	*Buxbaumia_aphylla*_scaffold-HRWG-2013254	No EF domain identified	TPC1b
	*Buxbaumia_aphylla*_scaffold-HRWG-2017281	No EF domain identified	TPC1b
	*Thuidium_delicatulum*_scaffold-EEMJ-2007306	IPR002048; PS50222; IPR011992; SSF47473	TPC1
	*Thuidium_delicatulum*_scaffold-EEMJ-2039009	IPR002048; PS50222; IPR011992; SSF47473	TPC1
	*Thuidium_delicatulum*_scaffold-EEMJ-2039845	No EF domain identified	TPC1b
	*Ceratodon_purpureus*_KAG0562970.1	IPR002048; PS50222; IPR011992; SSF47473	TPC1
	*Ceratodon_purpureus*_KAG0611548.1	IPR002048; PS50222; IPR011992; SSF47473	TPC1
	*Sphagnum_fallax*_Sphfalx04G006400.1	IPR002048; PS50222; IPR011992; SSF47473	TPC1
	*Sphagnum_fallax*_Sphfalx03G070700.1	No EF domain identified	TPC1b
	*Sphagnum_fallax*_Sphfalx16G086000.1	No EF domain identified	TPC1b
	*Sphagnum_fallax*_Sphfalx19G080400.1	IPR011992; SSF47473	TPC1b
	*Sphagnum_fallax*_Sphfalx16G017900.1	No EF domain identified	TPC1b
	*Takakia_lepidozioides*_scaffold-SKQD-2009335	IPR002048; PS50222; IPR011992; SSF47473	TPC1
	*Takakia_lepidozioides*_scaffold-SKQD-2080274	No EF domain identified	TPC1b
	*Takakia_lepidozioides*_scaffold-SKQD-2009261	No EF domain identified	TPC1b
Liverwort	*Marchantia_polymorpha*_Mapoly0002s0274.1	IPR011992; SSF47473	TPC1
	*Marchantia_polymorpha*_Mapoly0062s0036.1	No EF domain identified	TPC1b
	*Marchantia_polymorpha*_Mapoly0050s0061.1	No EF domain identified	TPC1b
Hornwort	*Nothoceros_vincentianus*_scaffold-TCBC-2005470	IPR002048; PS50222; IPR011992; SSF47473	TPC1

Streptophyte algae	*Chara_braunii*_GBG92126.1	IPR002048; cd00051; PS50222; SM00054; IPR011992; SSF47473; IPR018247; PS00018	
	*Chara_australis*_CL6562.Contig1-All.p1	IPR002048; SM00054; PS50222	
	*Klebsormidium_nitens*_kfl00001-0210-v1.1	IPR002048; PF13499; PS50222; IPR011992; SSF47473; IPR018247; PS00018	
	*Klebsormidium_nitens*_kfl00012-0290-v1.1	IPR011992; SSF47473	
	*Klebsormidium_nitens*_kfl00097-0180-v1.1	IPR002048; PS50222; IPR011992; SSF47473; IPR018247; PS00018	
	*Klebsormidium_nitens*_kfl00110-0290-v1.1	IPR002048; PS50222; IPR011992; SSF47473	
	*Klebsormidium_nitens*_kfl00312-0080-v1.1	IPR002048; PS50222; IPR011992; SSF47473	

Table S2: EF-hand domains in proteins from *A. thaliana* and in TPC1s from *P. patens*. The Arabidopsis protein list was taken from Day et al. [27]. The table is arranged by domains as identified by Proside local and we selected all potential EF-hand sequences with a *p*-value ≤ 1×10^{-5} ($p \leqslant 0.00001$).

ID Number	Number of EF hands	Domains	
		Start	End
AtTPC1_AT4G03560.1_EF1	1	322	357
AtTPC1_AT4G03560.1_EF2	1	363	398
PpTPC1.1.1_Pp3c1-6270V3.1_EF1	1	339	374
PpTPC1.1.2_Pp3c15-12230V3.1_EF1	1	331	366
PpTPC1.2.1_Pp3c1-28370V3.1_EF1	1	343	378
PpTPC1.2.3_Pp3c2-10230V3.1_EF1	1	373	408
PpTPC1.2.2_Pp3c14-20770V3.1_EF1	1	373	408
PpTPC1.3.1_Pp3c18-10190V3.1_EF1	1	341	376
PpTPC1.3.3_Pp3c21-11410V3.1_EF1	1	340	375
PpTPC1.3.2_Pp3c19-18680V3.1_EF1	1	342	377
PpTPC1.4.1_Pp3c3-16950V3.1_EF1	1	362	397
PpTPC1.1.1_Pp3c1-6270V3.1_EF2	1	380	415
PpTPC1.1.2_Pp3c15-12230V3.1_EF2	1	372	407
PpTPC1.2.1_Pp3c1-28370V3.1_EF2	1	384	418
PpTPC1.2.3_Pp3c2-10230V3.1_EF2	1	414	448
PpTPC1.2.2_Pp3c14-20770V3.1_EF2	1	414	448
PpTPC1.3.1_Pp3c18-10190V3.1_EF2	1	382	416
PpTPC1.3.3_Pp3c21-11410V3.1_EF2	1	381	415
PpTPC1.3.2_Pp3c19-18680V3.1_EF2	1	383	417
PpTPC1.4.1_Pp3c3-16950V3.1_EF2	1	403	437
AT1G02270.1	1	384	419
AT1G03960.1	1	381	416
AT1G05150.1	2	6	41
		600	635
AT1G05990.1	4	1	36
		37	72
		75	110
		113	148
AT1G09090.2	2	171	206
		215	250
AT1G12310.1	3	9	44
		80	115
		116	148
AT1G18210.1	4	19	54
		88	123

		55	85
		136	159
AT1G18530.1	4	3	38
		39	74
		78	113
		114	149
AT1G19230.2	1	268	303
AT1G20760.1	4	6	41
		42	75
		358	393
		395	427
AT1G21550.1	3	6	41
		85	120
		130	155
AT1G21630.2	4	8	43
		44	77
		457	492
		423	444
AT1G24620.1	4	33	68
		69	104
		106	141
		142	177
AT1G29020.2	2	318	353
		359	394
AT1G32250.1	4	12	47
		48	83
		91	126
		127	162
AT1G53210.1	2	299	334
		339	374
AT1G54450.1	2	182	209
		387	422
AT1G54530.1	1	20	55
AT1G62820.1	3	9	44
		80	115
		116	148
AT1G64060.1	1	264	299
AT1G64480.1	3	71	106
		108	143
		152	187
AT1G64850.1	1	66	101
AT1G66400.1	4	11	46

		47	82
		86	121
		122	157
AT1G66410.2	4	8	53
		54	89
		91	126
		127	159
AT1G73440.1	2	180	215
		216	251
AT1G73630.1	4	16	51
		85	120
		52	82
		121	156
AT1G76640.1	4	18	53
		54	89
		93	128
		129	159
AT1G76650.1	4	39	74
		75	110
		111	146
		147	177
AT2G03150.1	1	1270	1305
AT2G15680.1	4	46	81
		82	117
		129	152
		153	187
AT2G20800.1	1	384	419
AT2G27030.3	4	8	43
		44	79
		81	116
		117	152
AT2G27480.1	2	52	87
		121	156
AT2G32450.1	2	6	41
		595	630
AT2G34020.2	3	320	339
		357	392
		398	433
AT2G34030.1	2	296	331
		337	372
AT2G35800.1	2	339	355
		374	409

AT2G36180.1	4	1	31
		32	67
		72	107
		108	143
AT2G41090.1	4	8	43
		78	113
		44	73
		114	149
AT2G41100.1	6	8	43
		44	79
		97	132
		133	168
		187	222
		223	258
AT2G41110.2	4	8	55
		56	91
		93	128
		129	161
AT2G41410.1	4	66	101
		141	176
		103	138
		178	213
AT2G43290.1	4	61	96
		97	132
		139	174
		177	212
AT2G44310.1	2	20	55
		69	104
AT2G46600.1	1	74	109
AT3G01830.1	2	7	42
		116	146
AT3G03000.1	4	16	51
		52	87
		90	125
		126	161
AT3G03400.1	4	1	36
		37	72
		76	111
		112	137
AT3G03410.1	4	1	33
		34	69
		70	97

		98	131
AT3G03430.1	2	5	40
		43	75
AT3G05310.1	2	195	230
		316	351
AT3G07490.1	4	1	36
		37	72
		74	109
		112	147
AT3G10190.1	4	66	101
		103	138
		139	174
		176	209
AT3G10300.6	2	164	199
		230	265
AT3G17470.2	2	485	520
		522	554
AT3G18430.1	2	27	62
		91	126
AT3G20290.1	2	14	49
		52	83
AT3G22930.1	4	31	66
		67	102
		104	139
		140	173
AT3G24110.1	4	56	91
		92	127
		148	183
		196	222
AT3G25600.1	4	8	43
		44	79
		83	118
		119	154
AT3G29000.1	3	76	98
		122	157
		160	194
AT3G43810.2	4	8	43
		44	79
		81	116
		117	152
AT3G45810.1	1	209	240
AT3G47480.1	2	112	147

		149	183
AT3G50360.1	4	23	58
		59	94
		96	131
		132	167
AT3G50770.1	3	60	95
		96	131
		174	205
AT3G51920.1	4	8	43
		44	79
		81	116
		117	151
AT3G56800.1	4	8	43
		44	79
		81	116
		117	149
AT3G59440.1	4	47	82
		83	118
		119	154
		157	192
AT3G59450.1	1	111	146
AT3G59820.2	1	674	709
AT3G63150.1	2	195	230
		315	350
AT4G01420.1	3	66	101
		103	138
		147	182
AT4G03290.1	4	1	36
		37	72
		77	112
		115	150
AT4G05020.2	1	379	414
AT4G05520.1	2	15	50
		52	84
AT4G12860.1	4	1	36
		37	72
		74	109
		112	147
AT4G13440.2	2	18	53
		59	87
AT4G14640.1	4	9	44
		45	80

		82	117
		118	151
AT4G16350.1	3	76	111
		113	148
		157	192
AT4G17615.3	3	67	102
		104	139
		148	183
AT4G20780.1	3	25	60
		116	151
		154	189
AT4G25090.1	2	176	211
		220	255
AT4G25970.1	2	180	210
		211	246
AT4G26470.3	4	117	152
		153	188
		208	243
		244	279
AT4G26560.1	3	70	105
		107	142
		151	186
AT4G26570.2	4	64	82
		86	121
		123	158
		167	202
AT4G27280.1	1	72	107
AT4G27790.1	6	108	127
		141	163
		170	205
		221	244
		254	289
		293	328
AT4G28220.1	1	372	407
AT4G32060.1	4	243	278
		216	241
		329	364
		437	472
AT4G33000.1	3	113	148
		150	185
		194	229
AT4G37010.2	4	27	62

		63	98
		100	135
		136	171
AT4G38810.2	4	29	64
		94	110
		254	289
		300	335
AT5G04170.1	2	183	218
AT5G04170.1		249	284
AT5G06260.1	1	94	129
AT5G07320.1	4	33	68
		69	104
		105	135
		136	171
		221	256
AT5G08580.1	1	168	205
AT5G08580.1	5	216	245
		262	284
		299	334
		338	373
AT5G17470.1	4	1	33
		34	69
		73	108
		109	144
AT5G17480.1	2	5	40
		43	75
AT5G18580.1	3	186	221
		294	329
		369	404
AT5G24270.1	3	71	106
		108	143
		152	187
AT5G28850.2	3	182	209
		261	296
		387	422
AT5G28900.1	3	182	209
		261	296
		387	422
AT5G37770.1	4	13	48
		49	84
		90	125
		126	161

AT5G37780.3	5	8	25
		52	69
		70	105
		107	142
		143	175
AT5G39670.1	3	85	106
		132	167
		170	204
AT5G42380.1	4	45	80
		81	116
		119	154
		155	185
AT5G44090.2	3	220	247
		299	334
		425	460
AT5G44460.1	3	24	59
		107	142
		145	180
AT5G47100.1	3	67	102
		104	139
		148	183
AT5G47910.1	2	253	288
		297	332
AT5G49480.1	3	22	49
		52	87
		93	128
AT5G51050.1	4	47	82
		83	118
		119	149
		150	185
AT5G51060.1	2	226	261
		270	305
AT5G54130.2	1	336	371
AT5G54490.1	1	72	107
AT5G55990.1	3	82	117
		119	154
		163	198
AT5G57190.1	2	174	209
		210	245
AT5G60010.1	2	199	230
		239	274
AT5G61810.1	4	32	67

		68	103
		104	134
		135	170
AT1G18890.1	4	364	399
		400	435
		436	471
		472	507
AT1G35670.1	4	327	362
		363	398
		399	434
		438	468
AT1G50700.2	4	374	409
		449	471
		472	507
		508	542
AT1G61950.1	4	400	435
		436	471
		472	507
		512	542
AT1G74740.1	4	360	395
		396	431
		432	467
		468	503
AT1G76040.2	4	412	447
		448	483
		484	519
		520	554
AT2G17290.1	4	386	421
		422	457
		458	493
		497	527
AT2G17890.1	4	411	446
		448	483
		490	525
		528	555
AT2G31500.1	4	367	402
		403	438
		439	474
		478	513
AT2G35890.1	1	433	468
AT2G38910.1	4	435	470
		471	506

		507	542
		545	576
AT2G41860.1	4	355	390
		391	426
		427	462
		463	498
AT2G46700.1	2	446	481
		523	558
AT3G10660.1	4	487	522
		523	558
		593	628
		559	592
AT3G20410.1	4	392	427
		428	463
		464	499
		500	534
AT3G51850.1	4	355	390
		391	426
		427	462
		463	498
AT3G57530.1	4	364	399
		400	435
		471	506
		436	470
AT4G04700.1	4	332	367
		368	403
		404	439
		444	474
AT4G04710.4	4	346	381
		397	419
		420	455
		456	490
AT4G04720.1	4	380	415
		416	451
		452	487
		488	522
AT4G04740.2	3	369	404
		405	440
		441	476
AT4G09570.1	4	326	361
		362	397
		398	433

		437	467
AT4G21940.2	4	402	437
		438	473
		474	509
		510	544
AT4G23650.1	4	379	414
		415	450
		486	521
		451	485
AT4G35310.1	4	398	433
		434	469
		470	505
		509	539
AT4G36070.2	4	374	409
		411	446
		453	488
		491	518
AT4G38230.2	4	355	390
		391	426
		427	462
		466	496
AT5G04870.1	4	451	486
		487	522
		523	558
		561	592
AT5G12180.1	4	374	409
		410	445
		446	481
		485	516
AT5G12480.1	4	360	395
		396	431
		432	467
		469	504
AT5G19360.1	4	369	404
		405	440
		441	476
		480	511
AT5G19450.1	4	358	393
		395	430
		431	466
		467	502
AT5G23580.1	4	323	358

		359	394
		395	430
		434	464
AT5G66210.1	4	365	400
		402	437
		444	479
		482	509

Table S3. EF hand residues forming hydrogen bonds with calcium found in reported crystals of AtTPC1.

EF hand	Residues	PDBID	Reference
EF1	D335	7FHN, 7FHO	[36] (Ye *et al.*,2021)
		6E1M, 6E1N	[28] (Kintzer *et al.*,2018)
		5DQQ	[7] (Kintzer and Stroud, 2016)
		5E1J, 5TUA	[8] (Guo *et al.*, 2016)
	D337	7FHN, 7FHO	[36] (Ye *et al.*,2021)
		6E1M, 6E1N	[28] (Kintzer *et al.*,2018)
		5DQQ	[7] (Kintzer and Stroud, 2016)
		5E1J, 5TUA	[8] (Guo *et al.*, 2016)
	N339	6E1M, 6E1N	[28] (Kintzer *et al.*,2018)
		5E1J, 5TUA	[8] (Guo *et al.*, 2016)
	E341	7FHN, 7FHO	[36] (Ye *et al.*,2021)
		6E1M, 6E1N	[28] (Kintzer *et al.*,2018)
		5DQQ	[7] (Kintzer and Stroud, 2016)
		5TUA	[8] (Guo *et al.*, 2016)
	D343	5DQQ	[7] (Kintzer and Stroud, 2016)
		5TUA	[8] (Guo *et al.*, 2016)
	Q346	5DQQ	[7] (Kintzer and Stroud, 2016)
		5E1J, 5TUA	[8] (Guo *et al.*, 2016)
EF2	E374	6E1M, 6E1N	[28] (Kintzer *et al.*,2018)
		5DQQ	[7] (Kintzer and Stroud, 2016)
	D376	7FHN, 7FHO	[36] (Ye *et al.*,2021)
		6E1M, 6E1N	[28] (Kintzer *et al.*,2018)
	R379	6E1M,6E1N	[28] (Kintzer *et al.*,2018)
	T378	7FHN,7FHO	[36] (Ye *et al.*,2021)
	D380	7FHN, 7FHO	
	K382	7FHN, 7FHO	
	E387	7FHN, 7FHO	
