# Supplementary figures and images for: TPC1-Type Channels in Physcomitrium patens: Interaction between EF-Hands and Ca2+
Source: Plants (Basel). 2022 Dec 15;11(24):3527. doi: 10.3390/plants11243527 (PMC9783492; doi:10.3390/plants11243527)

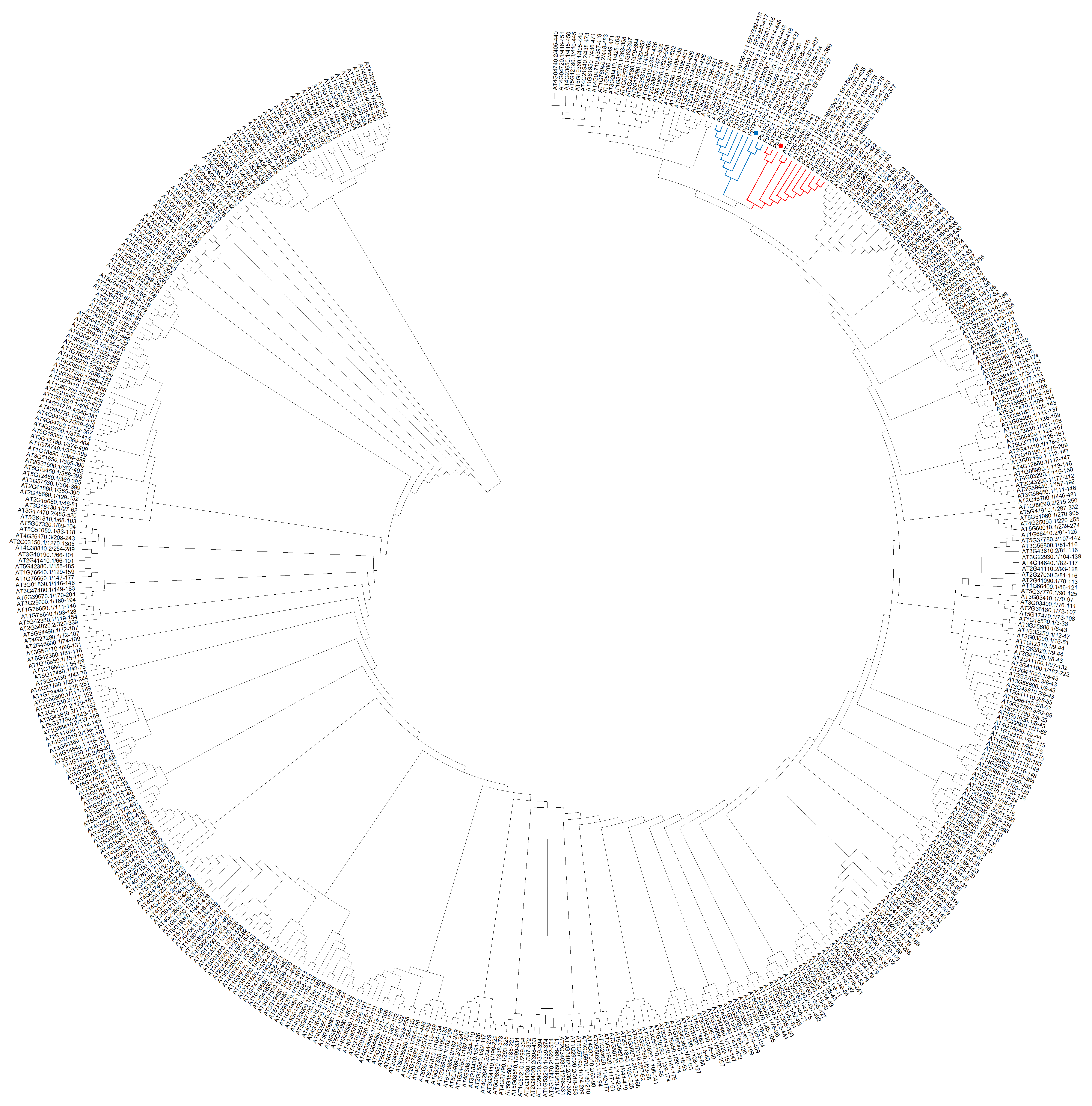

Supplement: Supplementary file 1 [file plants-11-03527-s001.zip › Figure_S3a.png]
